# A Reshaping Recovery: The Reverse Shoulder Arthroplasty Triumphs in Salvaging Chronic Four-Part Proximal Humerus Fractures

**DOI:** 10.7759/cureus.50363

**Published:** 2023-12-11

**Authors:** Hoe Teong Kee, Raymond D.K. Yeak, Khairil Anwar Ahmad Hanif, Siti Munira Seri Masran, Fahrudin Che-Hamzah

**Affiliations:** 1 Orthopaedic Surgery, Hospital Pengajar Universiti Putra Malaysia, Serdang, MYS; 2 Orthopaedic Surgery, Universiti Putra Malaysia, Serdang, MYS; 3 Orthopaedics and Traumatology, Hospital Pengajar Universiti Putra Malaysia, Serdang, MYS

**Keywords:** fixation failure, avascular necrosis (avn), open reduction internal fixation, reverse total shoulder arthroplasty, proximal humerus fracture

## Abstract

The aging population is witnessing a steady increase in the incidence of displaced proximal humerus fractures, particularly among elderly patients. Such fractures pose a significant challenge to orthopedic surgeons, given the complex interplay of factors involved, including fracture displacement, comminution, compromised bone quality, and the presence of concurrent medical comorbidities. While open reduction internal fixation (ORIF) remains a viable treatment option for these fractures, it is a technically demanding procedure associated with a high incidence of complications. Recently, reverse total shoulder arthroplasty (RTSA) with tuberosity repair has gained popularity as a successful approach for addressing such fractures. The present case report details a unique and complex case of a chronic four-part proximal humerus fracture, complicated by avascular necrosis of the humeral head, fracture non-union, and hardware penetration. The patient was successfully treated through a reverse shoulder arthroplasty procedure, highlighting the effectiveness of this surgical approach in such challenging scenarios. The advantages of RTSA in this context include the potential to address avascular necrosis, non-union, and hardware complications, as seen in our patient. Additionally, the procedure can restore functional independence and improve the overall quality of life in these challenging cases.

## Introduction

Proximal humerus fractures are among the most prevalent fractures in the elderly population, ranking third in frequency after hip and distal radius fractures, comprising nearly 10% of all fractures in this age group [1]. These fractures are primarily the result of low-energy traumas, such as falls. The aging of the population has led to a rising incidence of proximal humerus fractures, transforming it into a significant public health concern [1,2]. However, managing displaced fractures poses a considerable challenge due to issues like fracture displacement, comminution, poor bone quality, and the presence of concurrent medical comorbidities. It's worth noting that only about 20% of patients with proximal humerus fractures are considered candidates for surgery [2].

Recent years have seen notable shifts in the treatment of proximal humerus fractures, with a growing preference for internal fixation over hemiarthroplasty. Reverse shoulder arthroplasty (RSA) has gained prominence, particularly for displaced three- or four-part fractures in patients older than 70 years [1]. Traditional plates used for open reduction internal fixation (ORIF) were associated with high complication rates in osteoporotic bone due to hardware failure. However, advances in locking plate technology have improved the biomechanics of fracture fixation [1]. Nonetheless, ORIF remains linked to a high incidence of hardware penetration and related complications [1,3]. Agudelo et al. [1] reported a 13.7% complication rate due to hardware penetration in ORIF for proximal humerus fractures, and Brunner et al. [3] reported elevated complication rates, with the most common being primary hardware penetration followed by secondary penetration on follow-up. They also noted that older patients had over three times higher implant-related complications, largely attributed to bone quality.

In recent years, RSA has emerged as a favored option for acute proximal humerus fractures [4]. RSA alters the shoulder's center of rotation, lowers the humerus, and enhances the deltoid muscle's lever arm, resulting in improved forward elevation, even in cases of rotator cuff deficiency [5]. Published data [4-8] confirm that, compared to hemiarthroplasty, RSA offers significantly better and more consistent functional outcomes in terms of forward elevation, abduction, and constant score. The re-implantation of tuberosities around the prosthesis, followed by their anatomical healing, can also enhance rotations [9]. Even in cases where the repaired tuberosities do not fully heal, satisfactory functional outcomes are often achieved, in contrast to the major functional impairments observed after hemiarthroplasty. Additionally, the long-term risk of humeral component loosening is reduced through tuberosity repair, as it restores a metaphyseal bony region capable of withstanding the mechanical torque forces applied to the stem.

One of the primary causes of revision surgery after RSA is instability [10], which can be linked to errors in the height and/or version of the humeral stem. Therefore, a meticulous surgical approach is essential during the implantation of reverse shoulder prostheses to ensure consistent and dependable results.

In our report, we present a case involving a chronic four-part proximal humerus fracture complicated by avascular necrosis of the humeral head, fracture malunion, and hardware penetration. This complex case was successfully managed through an RSA procedure, emphasizing the effectiveness of RSA in addressing multifaceted challenges in proximal humerus fractures. This is to highlight that this is a complication that can be avoided if the predictive parameters for the failure of humeral head-preserving fracture fixation and anatomic hemiarthroplasty were carefully evaluated. Primary RSA for displaced proximal humerus fracture in the elderly was associated with better functional outcomes, lower complication, and revision rates than secondary RSA. Hence, primary RSA should be considered when surgical treatment of comminuted proximal humerus fracture is indicated in elderly patients.

## Case presentation

A 73-year-old gentleman with a medical history of diabetes mellitus, hypertension, dyslipidemia, and chronic kidney disease stage 3B was referred to a national university hospital due to fixation failure following a closed four-part proximal humerus fracture. The patient's injury occurred four months prior when he fell directly onto his right shoulder on a slippery floor. Radiographs revealed a four-part fracture of the right proximal humerus with significant displacement of the humeral head.

Initial treatment and clinical course

In a private hospital, ORIF was performed on the second day following the trauma using a right proximal humeral internal locking system (PHILOS) plate. The patient was discharged in stable condition. However, he continued to experience persistent shoulder pain, rating it at 8/10 on the pain scale, and developed shoulder stiffness. Notably, he denied any further trauma or falls after his discharge from the hospital. He denied fever and discharge from surgical wounds. During the examination of the right shoulder, we observed a well-healed anterolateral surgical scar, preserved deltoid muscle function, rotator cuff dysfunction, and no clinical signs of infection. Prominent screws were palpable, and there was tenderness over the fracture site. Shoulder range of motion was limited as follows: flexion 0-30 degrees, extension 0-15 degrees, abduction 0-45 degrees, adduction 0-20 degrees, external rotation 0 degrees, and internal rotation extended up to the sacral level. No neurological deficits were detected during the clinical evaluation and sensation over the regimental badge was intact.

**Figure 1:**
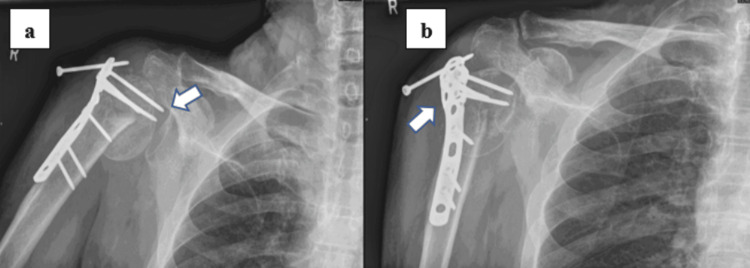
Radiographs of the right shoulder taken during the first clinic visit, four months after the surgery. (a) Anteroposterior radiograph of right shoulder demonstrating screw penetration (arrow) and collapse of the humeral head. (b) Lateral radiograph of the right shoulder shows a locking screw backout (arrow) and prominent fracture line.

Subsequent radiographs of the right shoulder during the clinic visit revealed loosening of the proximal locking screw, penetration of screws into the glenohumeral joint, collapse of the humeral head, and a prominent fracture line (Figures 1a-1b). Laboratory blood tests revealed the following results: total white blood cell count (TWBC) of 6.6, a C-reactive protein (CRP) level of 0.1, and an erythrocyte sedimentation rate (ESR) of 30 indicated that inflammatory markers were within normal ranges. The probable mechanisms of failure of internal fixation in this patient were advancing age, osteoporosis, four-part-fracture, medial metaphyseal comminution, medial metaphyseal head extension, medial hinge displacement, and displacement of tuberosities with respect to the humeral head. After careful pre-operative planning, the decision was made to perform a single-stage salvage procedure by removing the right PHILOS plate and replacing it with a right RSA.

Surgical procedure

**Figure 2:**
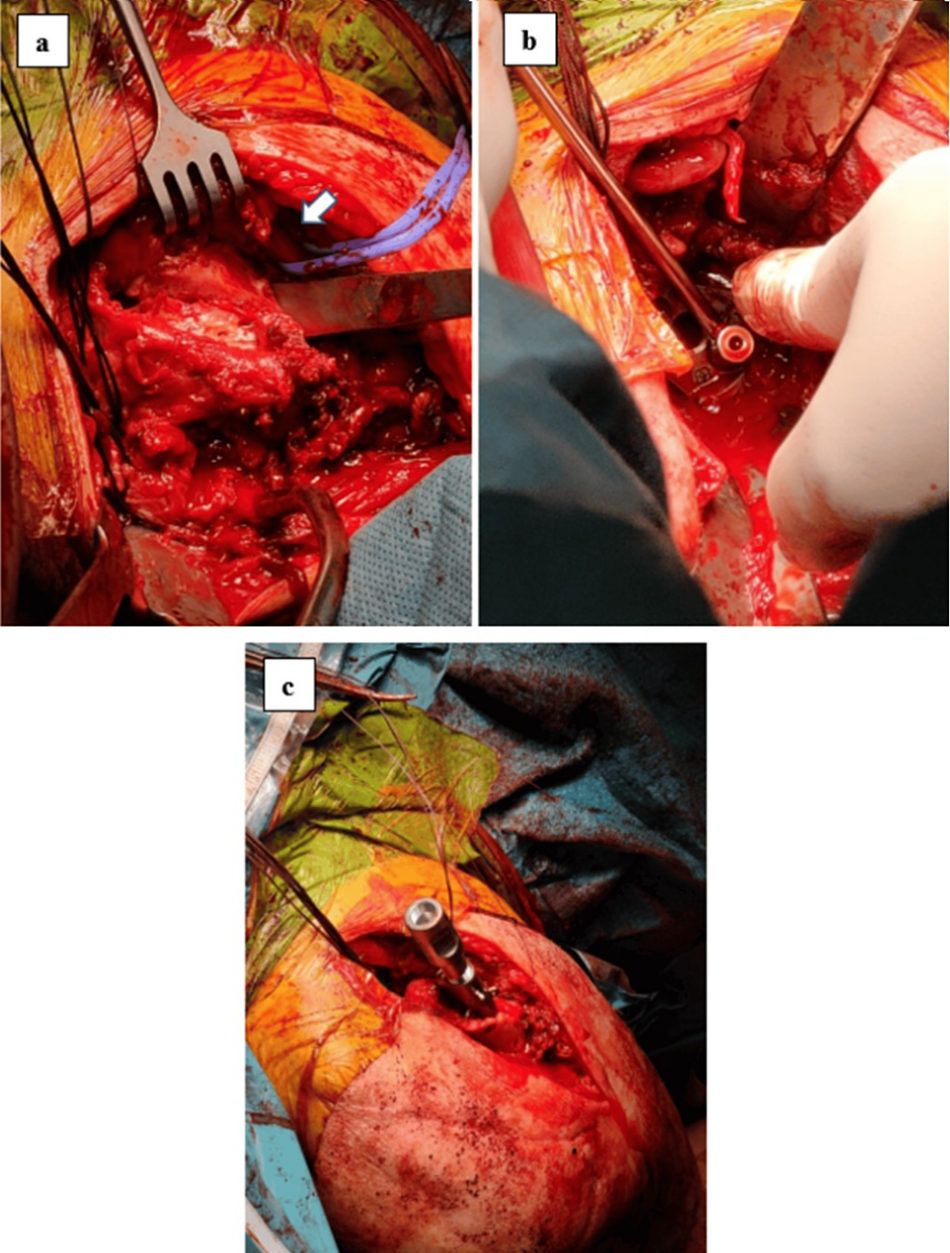
Intra-operative clinical pictures a) This clinical picture depicts a window created between the greater tubercle and the lesser tubercle with the assistance of an osteotome. The long head of the biceps tendon has been retracted (indicated by the arrow), and each tuberosity and cuff insertion were secured with sutures to facilitate control of these fragments. b) In this clinical picture, you can observe the baseplate being implanted into the prepared glenoid fossa. c) This clinical picture illustrates the preparation and implantation of the humeral stem into the humerus with the correct orientation.

The patient underwent surgery after thorough pre-operative medical optimization to address his underlying medical comorbidities. He was positioned in a beach-chair stance, and intra-operative fluoroscopy was employed to facilitate the acquisition of anterior-to-posterior and axillary images of the right shoulder. Following the induction of general anesthesia, 1.5g of intravenous cefuroxime was administered. The right shoulder was then prepared and draped, and a timeout was announced. In order to facilitate surgical exposure, implant removal, and arthroplasty procedure, a new deltopectoral incision was made, and the deltoid and pectoralis major muscles were retracted laterally and medially, respectively. At this stage, the biceps tendon was identified and tenotomized at the base of the superior labrum. The anatomy of the proximal humerus was distorted, tuberosities were malunited, and the humeral head was non-union with varus collapsed after removal of the PHILOS plate and locking screws. In view of distorted anatomy, an intra-operatively image-intensifier was used to identify the location for osteotomy in between greater and lesser tuberosity to create a window and subsequently remove the non-union humeral head fragment with the cork-screw humeral head extractor (Figure 2a). Each tuberosity and cuff insertion was tagged with sutures to enable control of these fragments. The humeral head was covered with joint capsules and scar tissues and the removal of scar tissues and capsules was done with the aid of diathermy. By using this deltopectoral approach, there is no difficulty in the extraction of the humeral head. There were no arthritic changes of glenoid fossa articular cartilage and no fracture of glenoid was seen. The glenoid was prepared, the central peg hole was drilled, and the trajectory of the other baseplate screw was estimated. A baseplate with a central peg was implanted to provide more flexibility regarding the positioning of the surrounding baseplate screw (Figure 2b). The glenoid baseplate coverage was adequate with stable fixation with one central peg screw and two peripheral screws. Right glenoid anatomical size 2 cementless and glenoid hemisphere cap size 40mm were inserted. Subsequently, the humerus was reamed and broached. The trial humeral stem was inserted, and a trial reduction was performed using various polyethylene sizes (Figure 2c).

**Figure 3:**
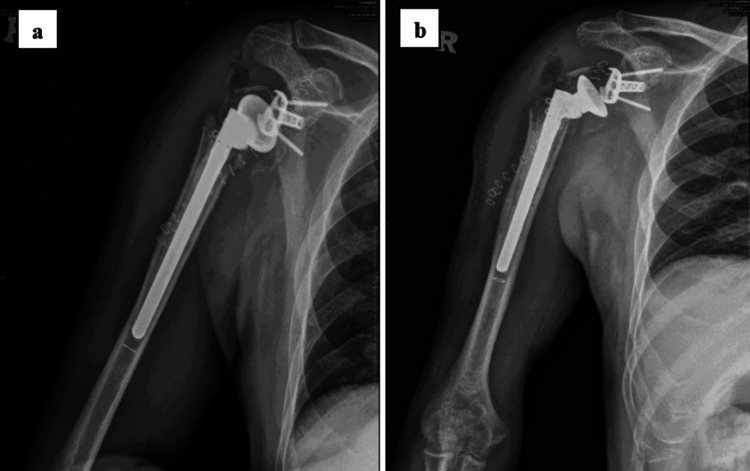
Post-operative radiographs of the right shoulder reveal a secure fixation of reverse shoulder arthroplasty (Implantcast™, Germany) with adequate cement mantle implantation and a radiolucent glenoid hemisphere. Implant used (Implantcast™, Germany). Cementless right glenoid baseplate anatomical size 2. Glenoid hemisphere cap size 40mm. Right cemented humerus shaft stem size 10mm in diameter and 120mm in length. Two cancellous peripheral screws are 4.2mm and 34mm in length. One central peg screw is 6.5mm and 25mm in length. (a) Lateral view; (b) Anteroposterior view

A threaded rod was inserted into the trial and aligned with the axis of the forearm. The humeral stem was positioned in 30° of retroversion. Height was established by placing the articular surface of the prosthetic head at the midpoint of the glenoid fossa, and with gentle traction, the prosthetic head remained within the glenoid fossa. This is to allow for appropriate deltoid muscle moment arm. Following the reduction, a radiographic assessment of the tuberosity position and alignment was conducted. Image intensifier images confirmed near-anatomic alignment, and the position was accepted. A cement restrictor was inserted and cementation was done with finger packing. Right cemented humerus shaft stem size 10mm in diameter and 120mm in length was inserted. The tuberosities were secured in place with fiber wire suture through multiple cerclage stitches, following the technique described by Frankle and Mighell [11], and further augmentation with autologous bone graft harvested from the humeral head to close up the rotator interval to allow for union of tuberosities. Post-operative radiographs of the right shoulder demonstrated secure fixation and proper alignment of the prosthesis (Figures 3a-3b). The procedure proceeded without complications, and the patient was discharged three days post-surgery.

Initial recovery and follow-up

Following the surgery, the patient was placed in a shoulder brace and instructed to engage in exercises to enhance elbow, wrist, and hand mobility for a duration of two weeks. Sutures were removed during the clinic follow-up at the end of two weeks, revealing a well-healed wound with no signs of infection. The patient was treated under a multidisciplinary team approach for his underlying chronic kidney disease and osteoporosis. Calcium supplements were tab calcium carbonate 500mg twice daily and tab calcitriol 0.25mcg daily.

Rehabilitation progress

After the initial two-week period, the patient's rehabilitation regimen included passive range of motion exercises. At the six-week mark, the shoulder brace was removed, allowing for active-assisted motion and more aggressive stretching.

Three months post-surgery assessment

**Figure 4:**
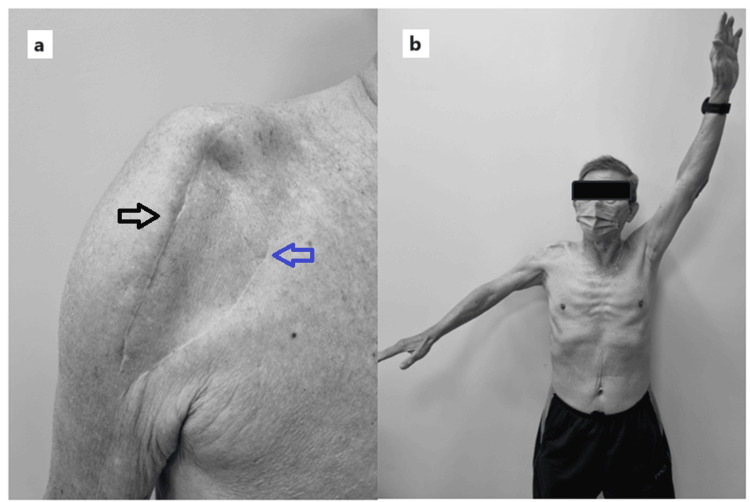
Clinical pictures of the patient at three months post-operative a) Clinical picture shows the incision made over the right shoulder. The anterolateral incision for primary open reduction and internal fixation (ORIF) (black arrow) and deltopectoral incision for salvage reverse shoulder arthroplasty (RSA) surgery (blue arrow). 2) Clinical picture shows active shoulder abduction of 70 degrees over right shoulder post salvage RSA procedure.

**Figure 5:**
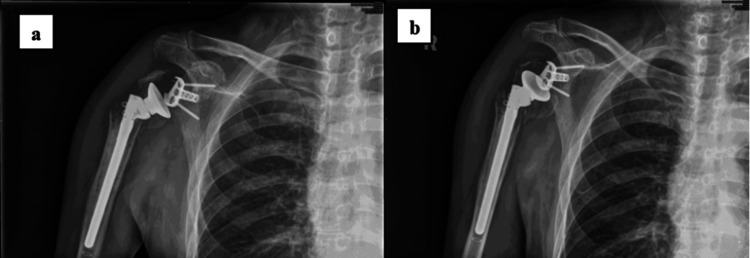
Radiographs of the right shoulder taken three months after reverse shoulder arthroplasty show callus formation in the tuberosities with no evidence of prosthesis loosening. (a) Anteroposterior view; (b) Lateral view

At three months following the surgery, the patient's progress was as follows: active abduction to 70 degrees, active flexion to 70 degrees, external rotation to 15 degrees, and internal rotation extended to his gluteal region (Figures 4a-4b). Functional outcomes score American Shoulder and Elbow Surgeons score (ASES) improved from 27/100 pre-operative to 77/100 three months post-operative. Notably, the patient experienced excellent pain control and displayed no signs of instability or any complications related to the implant. Radiographs of the right shoulder taken three months after RSA show callus formation in the tuberosities with no evidence of prosthesis loosening (Figures 5a-5b).

## Discussion

The optimal treatment for displaced proximal humerus fractures has been a topic of significant debate within the medical community. Several well-documented treatment options exist in the literature, including nonoperative management, hemiarthroplasty, RSA, open reduction and internal fixation, and proximal humeral nailing [11-13]. The decision to opt for surgical intervention in proximal humerus fractures typically arises when the viability of the humeral head is in question. Indications for surgical intervention include gross displacement of the humeral head, the absence of a medial hinge, and a medial calcar measuring less than 8mm in size [14].

Challenges and complications

Studies, such as the one conducted by Konighausen et al. [15], have examined patients who underwent ORIF for predominantly three- and four-part fractures. They reported an overall complication rate of 23%, with humeral head osteonecrosis being the most common complication, followed by hardware penetration. Salvage procedures for chronic four-part proximal humerus fractures are technically challenging and require meticulous pre-operative planning.

Indications for RSA

RSA is recommended for patients older than 70 years who have a three- or four-part displaced fracture and are at high risk of developing avascular necrosis of the humeral head, possess poor-quality comminuted tuberosities, or have a pre-existing rotator cuff tear [5]. It's important to note that RSA is not the first-line treatment for young and active patients. In elderly patients with recent proximal humerus fractures, RSA consistently provides not only globally satisfactory outcomes but also outcomes that are reliable and sustained over time. Hemiarthroplasty, in contrast, tends to lack reproducible and reliable outcomes, particularly in cases of tuberosity non-union or migration [16].

Optimal surgical technique for tuberosity healing

The successful anatomical union of the greater tuberosity is a critical goal of the procedure, as it contributes to satisfactory and sustained outcomes over time. Achieving this involves using a rigorous surgical technique that combines a specific non-filling stem with bone grafts and robust tuberosity repair [2]. Re-implanting the tuberosities around the prosthesis is a pivotal step that enhances functional outcomes by restoring rotation ranges and reducing the risk of complications, such as prosthesis instability and humeral component loosening. Notably, even when the repaired tuberosities do not fully heal, functional outcomes remain satisfactory, in contrast to the significant functional impairments often observed after hemiarthroplasty.

Contraindications and considerations

Contraindications for RSA include pre-existing or concomitant axillary nerve injury, concomitant fractures of the scapular spine or acromion that may be displaced due to increased tension in the deltoid muscle, and concomitant glenoid fractures that may impede the placement of a glenoid baseplate [17]. Despite the advanced age of the population, the immediate mortality and loss of self-sufficiency following RSA appear to be considerably less than those associated with hip arthroplasty for femoral neck fractures. As a result, elderly patients can anticipate many years of life with their RSA, and outcomes tend to remain stable over time.

Optimal procedure goals

The primary objective of the procedure is to achieve the healing of the tuberosities in their anatomical positions [18]. To this end, an optimal operative technique is crucial. This technique should include the use of a non-filling fracture stem, achieving anatomical reduction and secure fixation of the tuberosities, positioning the glenoid component at the lower rim of the glenoid cavity without superior tilt, post-operative immobilization (preferably in a neutral rotation or slight abduction position), and the option of deferred rehabilitation therapy or the introduction of gentle pendulum exercises immediately post-surgery [18].

## Conclusions

In conclusion, the successful management of chronic four-part proximal humerus fractures in elderly patients hinges on a comprehensive and tailored approach, with RSA proving to be a valuable tool in restoring function and quality of life in the salvage of these cases.
